# Hypertension and immune activation in antiretroviral therapy naïve people living with human immunodeficiency virus

**DOI:** 10.1186/s12879-024-09548-x

**Published:** 2024-06-24

**Authors:** Tosi M. Mwakyandile, Grace A. Shayo, Philip G. Sasi, Ferdinand M. Mugusi, Godfrey Barabona, Takamasa Ueno, Eligius F. Lyamuya

**Affiliations:** 1 Department of Clinical Pharmacology, School of Biomedical Sciences, Campus College of Medicine, Muhimbili University of Health and Allied Sciences (MUHAS), Dar es Salaam, Tanzania; 2 Department of Internal Medicine, School of Clinical Medicine, Campus College of Medicine, Muhimbili University of Health and Allied Sciences (MUHAS), Dar es Salaam, Tanzania; 3 Division of Infection and Immunity, Joint Research Center for Human Retrovirus Infection, Kumamoto University, Kumamoto, Japan; 4 Collaboration Unit for Infection, Joint Research Center for Human Retrovirus Infection, Kumamoto University, Kumamoto, Japan; 5 Department of Microbiology and Immunology, School of Diagnostic Medicine, Campus College of Medicine, Muhimbili University of Health and Allied Sciences (MUHAS), Dar es Salaam, Tanzania

**Keywords:** Hypertension, Monocyte activation, Lymphocyte activation, HIV, Antiretroviral therapy

## Abstract

**Background:**

The pathogenesis of hypertension (HTN) in people living with HIV/AIDS (PLHIV) is complex and remains not fully understood. Chronic immune activation (IA) is postulated to be one of the culprits. This notion is derived from studies in HIV-uninfected populations and/or animals while data on HTN and how it relates to IA in PLHIV remains scarce. We determined the relationship between HTN and IA among antiretroviral therapy (ART) naïve PLHIV.

**Methods:**

We analysed baseline data of 365 out of 430 clinical trial participants whose main aim was to investigate the effect of low-dose aspirin on HIV disease progression in PLHIV starting ART. Soluble CD14 (sCD14), T cells co-expressing CD38 and HLA-DR, and PD-1 were the IA and exhaustion markers, respectively studied and were analysed by flow cytometry. Mann-Whitney U-test was used for comparison of the markers by HTN status. A robust Poisson regression model was used to determine the predictors for HTN.

**Results:**

A quarter of the 365 were hypertensive (25.3%, 95% CI 20.9–29.8%), and, had higher median (IQR) body mass index (kg/m^2^) (23.4 (19.6, 28.0) versus 21.9 (19.3, 25.1)) and lower median (IQR) estimated glomerular filtration rate (mL/min/1.73m^2^) (101.2 (79.4, 126.9) versus 113.6 (92.7, 138.8)) than normotensive participants (*p* < 0.05). Participants with HTN had higher median frequencies of all markers of IA and exhaustion but lower sCD14 (*p* > 0.05). None of these markers significantly predicted the occurrence of HTN.

**Conclusion:**

Studied markers of IA and exhaustion were higher in PLHIV with HTN than those without but were unpredictive of HTN. Larger multicentre studies with a wider range of markers are needed to confirm the role of IA in HIV-associated HTN.

**Supplementary Information:**

The online version contains supplementary material available at 10.1186/s12879-024-09548-x.

## Introduction

Non-AIDS complications such as cardiovascular diseases (CVDs) have become important causes of morbidity and mortality among people living with HIV/AIDS (PLHIV) in the antiretroviral therapy (ART) era, globally and in Africa [1–3].

Of the CVDs, hypertension (HTN) is of paramount importance not only as a CVD but also as a topmost risk factor for other CVDs. In PLHIV, HTN is an important contributor to cardiovascular (CV) illness and deaths. Western literature shows that the risk for CV events and deaths from all causes is higher in adult hypertensive PLHIV than in adult non-hypertensive PLHIV and adult hypertensives in the general population [4–6]. Furthermore, the risk for incident acute myocardial infarction (AMI) is higher in hypertensive PLHIV than in hypertensive HIV- uninfected population [4]. However, there is limited data on the impact of HTN on CVDs and mortality among PLHIV in the African population. A study conducted in Kenya among PLHIV revealed that men with HTN, but without advanced HIV disease, had a higher mortality risk compared to HIV- infected men who were not hypertensive [7].

Data from many parts of the globe including Africa show that the prevalence of HTN in PLHIV is high and is increasing where up to a quarter of PLHIV irrespective of antiretroviral therapy (ART) status have HTN [8]. Although the prevalence of HTN in HIV-uninfected population and/or ART exposed PLHIV is higher than in ART naïve PLHIV [8–12], there is evidence to show that HTN also is a problem in ART naïve PLHIV. Indeed, a study conducted in Cameroon has shown HTN to be more prevalent in ART naïve PLHIV than in ART-exposed PLHIV and the HIV-uninfected population [9]. A recent review has reported that 12.7% of ART naïve PLHIV in the world have HTN [8]. In Sub-Saharan Africa (SSA), prevalence of HTN, up to 41%, in ART naïve PLHIV has been reported [9]. Tanzania too, has a high burden of HTN among ART naïve PLHIV ranging from 5.3 to 24.8% [10, 11, 13–16].

Many factors contribute to HIV-associated HTN, and these may partly explain the conflicting data on the prevalence of HTN in the literature between ART-exposed and ART naïve PLHIV. ART may have a role in the aetiology of HIV-associated HTN. For example, dolutegravir (DTG)-based regimens (which are currently the first-line choice for HIV- infection in most of SSA including Tanzania) have been associated with HTN [17, 18]. This is alarming because ART naïve PLHIV are initiated on a lifelong treatment with ART and hence will be likely faced with more HTN in the future.

The underlying mechanism behind HIV-associated HTN may be complex and remains poorly understood. Apart from ART, pathophysiologic mechanisms for HIV- associated HTN including microbial translocation, immune suppression/ reconstitution, and chronic immune activation (IA) [9, 19] have been postulated.

Chronic IA appears to play a central role in the pathogenesis of HIV- associated HTN. ART naïve PLHIV have higher levels of chronic IA than HIV-uninfected and ART exposed population [20, 21]. Furthermore, the levels of IA are higher in ART naïve and do not normalize even with successful ART [20–24], underscoring a need for an anti-inflammatory drug. The exaggerated chronic IA in ART naïve PLHIV and the residual chronic IA in ART-treated PLHIV may be responsible for non-AIDS complications like HTN.

Of the immune cells, it appears that the activation of T-cells and monocytes plays a significant role in the development of CVDs including HTN. However, there is a paucity of data on the relationship between IA and HTN in PLHIV reported in the literature, and most reported data were obtained from studies in the general population and/or animal models of HTN [9, 25]. Furthermore, most studies of HIV and IA/ inflammation have not looked at IA specifically as it relates to HTN [9, 25]. In view of this knowledge gap, we studied some of the cellular and plasma markers of IA and their relationship to HTN in ART naïve PLHIV. The knowledge of IA is important in HIV-associated HTN as it may offer new potential therapeutic targets for the prevention and improvement in the clinical management of HTN in PLHIV.

## Methods

### Study design, study setting, study population

This article presents the findings of the analysis of baseline data from a clinical trial whose main aim was to investigate the effect of low-dose aspirin (ASA) on HIV disease progression in PLHIV starting ART. The trial was registered in both the Pan African Clinical Trial Registry (PACTR202003522049711) and ClinicalTrials.gov (NCT05525156). An elaborate methodology of the trial has been previously described [26]. Briefly, the trial participants were recruited from three different care and treatment centres (CTCs). The selected CTCs, situated in two regional referral and one district public hospitals in Dar es Salaam, Tanzania’s largest city and financial centre, contribute to catering to a population where HIV prevalence among adults stands at 4.2%. The services in these CTCs are coordinated by the Tanzanian government through the National AIDS Control Programme and supported by Management and Development for Health, a non-profit non-governmental organisation. These CTCs specialise in HIV outpatient care and provide free HIV/AIDS testing and counselling; treatment, and monitoring. The CTCs are equipped with staff, including clinicians, nurses, pharmacists, laboratory officers, and counsellors, who are trained in HIV care. Being situated in district and regional referral hospitals these CTCs serve individuals from various socioeconomic backgrounds within their districts and neighbouring areas outside Dar es Salaam.

Recruitment was between March 2020 and June 2022 with a three-month temporary suspension due to the COVID-19 pandemic. The inclusion criteria involved being newly diagnosed with HIV, ART naïve, starting ART at the time of enrolment, aged 18 years or older, and being willing to participate for six consecutive months. Exclusion criteria included asthma, pregnancy, bleeding predisposition, use of antithrombotic drugs, use of trial-prohibited drugs (see supplementary file 1), peptic ulcer disease, ASA intolerance or allergy, and/or severe kidney disease (estimated glomerular filtration rate (eGFR) < 30 mL/min/1.73m^2^).

### Data collection

Participants who fulfilled eligibility criteria underwent interviews, physical examinations, and data recording of their sociodemographic and clinical information. Details of age, alcohol consumption, cigarette smoking, individual history of CVDs and diabetes mellitus (DM), and family history of CVDs were recorded. Current and previous medication history including use of antidiabetics, antihypertensives, and antidyslipidaemics was also documented. Body weight was measured in kilograms using a digital weighing scale (Health O Meter, 500KL, China), and body height was measured in centimetres using a stadiometer (Health O Meter, 500KL, China). These measurements were used to calculate the body mass index (BMI) [27]. Blood pressure (BP) measurements were taken from the left arm while sitting for each participant using a digital sphygmomanometer (Yuwell YE660D, Jiangsu Province, China). Two readings were recorded, with a time gap of five to ten minutes, and the average of the two readings was calculated and used as the participant’s BP [28].

### Laboratory procedures

Each participant provided a total of 20mL of non-fasting antecubital venous blood sample that was aliquoted thrice and transported, in a cool box, to the Muhimbili University of Health and Allied Sciences (MUHAS) laboratories. At MUHAS, two 4mL aliquots: one for full blood picture (FBP) and CD4, and another for serum creatinine and lipid profile, were sent to the MUHAS Clinical Research Laboratory (MCRL) where 50µ of the aliquot in K2 EDTA vacutainer tube underwent FBP analysis (Sysmex analyser, Sysmex Corporation, Japan) while the remaining volume of this sample was kept in the cool box and later transported to the Infectious Disease Centre laboratory (IDC) within Dar es Salaam for CD4 count (FACSPresto; BD Biosciences, San Jose, California, USA). The aliquot for serum creatinine and lipid profile was centrifuged to obtain serum and analysed (creatinine, total cholesterol (TC), High-density lipoprotein cholesterol (HDL-C), and Triglycerides (TG) (COBAS Integra 400 Plus, Roche Instruments Centre AG, Rotkreuz, Switzerland). The sample processing and analysis window for FBP, serum creatinine and lipid profile was six hours and 24 h for CD4 count. The 12mL aliquot (sterile K2 EDTA tubes) was sent to the Immunology laboratory (IL) where it was immediately centrifuged at 1500 times gravity (x g) for 10 min at minimum acceleration and deceleration to obtain plasma and cellular sediment. The obtained plasma was aliquoted to 1.5 mL and 4.5 mL and immediately stored at -80 °C. From the cellular sediment, peripheral blood mononuclear cells (PBMCs) were isolated by density gradient centrifugation using Ficoll-Paque Plus media solution (GE Healthcare Life Sciences Inc., Chicago, Illinois), re-suspended in 2 mL of Fetal Cow Serum containing 10% Dimethyl Sulfoxide and stored in liquid nitrogen. At the end of the study, the 1.5 mL plasma was sent to the Muhimbili National Hospital Central Pathology laboratory for viral load analysis. The 4.5 mL plasma and the PBMCs were shipped, in dry ice, to the Joint Research Center for Human Retrovirus Infection laboratory at Kumamoto University, Kumamoto, Japan where they were stored (plasma at -80 °C, PBMCs in liquid nitrogen) until analysis for monocyte activation marker (soluble CD14), platelet activation marker (soluble P-selectin) and T lymphocyte activation.

In our study, serum creatinine was used for calculating eGFR (Modification of Diet in Renal Disease study equation) that was used for staging chronic kidney disease (Kidney Disease Improving Global Outcomes staging system [29]. Low-density lipoprotein cholesterol (LDL-C) was estimated by the Friedewald equation [30].

### Soluble CD14 (sCD14) and soluble P-selectin (sP-selectin) analyses

Thawed plasma were diluted at 1:50 for sP-selectin and 1:1000 for sCD14 and respective biomarkers measured using a customised BD^TM^ Cytometric Bead Array kit (BD Biosciences, San Jose, California, USA) according to the manufacturer’s instruction manual. Standards from lowest to highest concentrations followed by test samples were acquired by flow cytometry (BD FACSCanto™ II, BD Biosciences, San Jose, California, USA) using FACSDiva software (BD Biosciences, San Jose, California, USA) at 400 events before analysis by flow cytometric analysis program array software (Soft Flow Hungary Ltd., Hungary).

### PBMCs analysis for markers of T lymphocyte activation

**Fig. 1:**
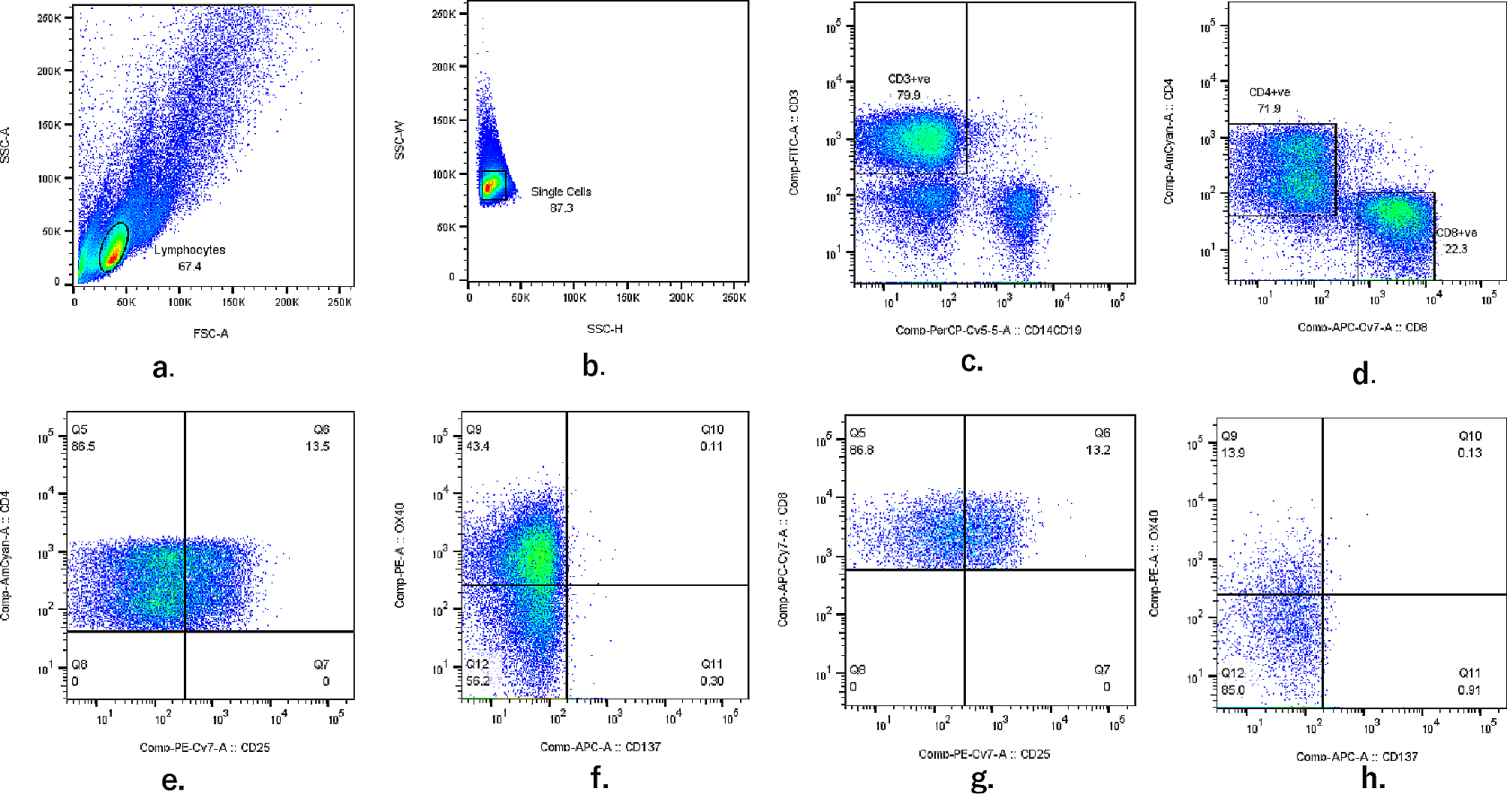
Expression of activation (CD38 and HLA-DR) and exhaustion (PD-1) markers on CD4 + and CD8 + T cells. (**e**). Activation marker to define PD-1 + CD4 + T cells. (**f**). Activation marker to define CD38 + HLA-DR + CD4 + T cells. (**g**). Activation marker to define PD-1 + CD8 + T cells. (**h**). Activation marker to define CD38 + HLA-DR + CD8 + T cells

Liquid nitrogen-frozen PBMCs were thawed, stained in the dark with diluted (1:100) antibodies: CD3 FITC, CD14 PerCP, CD19 PerCP, CD8 APCcy7, CD4 BV510, CD38 PE, PD-1 PEcy7 and HLA-DR APC and fixed with 1% paraformaldehyde. About 100,000 events were acquired for each sample using FACSDiva software (BD Biosciences, San Jose, California, USA) by flow cytometry (BD FACSCanto™ II, BD Biosciences, San Jose, California, USA). Frequencies of activated (CD38 + HLA-DR+) and exhausted (PD-1+) T lymphocytes (CD4 + and CD8+) were determined by gating based on isotype controls using FACSDiva software (BD Biosciences, San Jose, California, USA) (Fig. 1). Data were obtained on FlowJo™ version 10.8.2 software (TreeStar, Ashland, Oregon).

### Definitions of variables

Hypertension was defined as the individual’s systolic BP (SBP) of ≥140 mmHg and/or the individual’s diastolic BP (DBP) of ≥ 90 mmHg and/or history of HTN and/or current or past use of antihypertensives [31]. The age considered at risk for HTN was 45 years or older for men and 55 years or older for women [32]. Alcohol consumption was defined as the current or past regular use of alcohol. Cigarette smoking was defined as the current or past regular smoking of cigarettes. Diabetes mellitus (DM) was defined as a history of DM and/ or current or past use of antidiabetic medication. A history of CVDs was defined as a participant’s previous occurrence of stroke and/or MI [32]. Family history of CVDs was defined as the presence of HTN and/or stroke and/or MI in the immediate relatives of the participant [32]. Overweight was defined as a BMI of 25.0 to 29.9 kg/m^2^ and obesity as a BMI of ≥ 30.0 kg/m^2^. Dyslipidaemia was defined as non-fasting serum TC ≥5.17 mmol/ L and/or LDL-C ≥ 3.36 mmol/ L and/or TG ≥ 1.70 mmol/ L and/or current use of antidyslipidaemics regardless of sex and/or HDL-C <1.03 mmol/ L for men or HDL-C <1.29 mmol/ L< for women [32].

### Data management and statistical analysis

Data in case report forms (CRFs) were compared to data on the source document for accuracy and completeness. Double data entry, verification and cleaning were done on a password-secured computer followed by analysis on statistical software for social sciences (SPSS) for Windows version 26 (Inc., Chicago, Illinois). Study participants’ characteristics were described using descriptive statistics. Mean ± standard deviation (SD) or median (interquartile range (IQR)) were used to present continuous variables based on the distribution of the data. Frequencies and percentages were used to express categorical variables.

Mann-Whitney U test was used to compare levels of IA and exhaustion; and platelet activation between participants with HTN and those without HTN. A robust Poisson regression model was used to examine the predictors for HTN because, in this study, the prevalence of HTN (25.3%) was high (> 10%). The variables that had a *p*-value < 0.2 in the univariable analysis were included in the multivariable analysis. A *p*-value of < 0.05 in the multivariable analysis was considered statistically significant.

## Results

### Socio-demographic and clinical characteristics

Three hundred sixty-five participants who had at least one marker of the immune or platelet activation measured out of 430 total clinical trial participants were included in this analysis. Majority of hypertensive participants were self-employed and living with partner compared to the normotensive participants (*p* < 0.05). More normotensive participants had a history of bacterial infection at enrolment than hypertensive participants (*p* = 0.009). Median BMI was higher among hypertensive participants than among normotensive participants (*p* = 0.006). Median eGFR was lower among hypertensive participants than among normotensive participants (*p* = 0.003). Participants with HTN had higher median (IQR) frequencies of markers of T cell activation and exhaustion compared to non-hypertensive participants, but these were not statistically significantly different. Site of recruitment, level of education and other clinical characteristics were comparable between the two groups of participants (Table 1).

**Table 1:** Socio-demographic and clinical characteristics of HIV-infected treatment naïve individuals initiating ART overall (*N = 363), and by hypertension status

Variable		Total participants	Hypertensive	Normotensive	*p*-value
Median (IQR) age (years)		37 (28, 45)	38 (33, 45)	36 (27, 45)	0.06
Male sex, *n* (%)		129 (35.5)	39 (42.4)	90 (33.2)	0.11
Employment status, *n* (%) *N* = 362					
	Employed	57 (15.7)	6 (6.6)	51 (18.8)	**0.004**
	Self employed	230 (63.5)	70 (76.9)	160 (59.0)	
	Unemployed	75 (20.7)	15 (16.5)	60 (22.1)	
Marital status, *n* (%) *N* = 362					
Living alone		202 (55.8)	40 (43.5)	162 (60.0)	**0.006**
Living with partner		160 (44.2)	52 (56.5)	108 (40.0)	
^a^Risky Age for CVDs, *n* (%)					
	Yes	59 (16.3)	19 (20.7)	40 (14.8)	0.19
Cigarette smoking, *n* (%)					
	Ever smoked	57 (15.7)	11 (12.0)	46 (17.0)	0.25
Alcohol consumption, *n* (%)					
	Ever consumed	173 (47.7)	42 (45.7)	131 (48.3)	0.66
Diabetes Mellitus, *n* (%)					
	Yes	3 (0.8)	2 (2.2)	1 (0.4)	0.16
Family history of CVDs, *n* (%)					
	Yes	49 (13.5)	13 (14.1)	36 (13.3)	0.84
Dyslipidaemia, *n* (%) *N* = 163					
	Yes	144 (88.3)	47 (90.4)	97 (87.4)	0.58
History of bacterial infection at enrolment, *n* (%)					
	Yes	69 (19.0)	9 (9.8)	60 (22.1)	**0.009**
History of use of anti-inflammatory drug within past month, *n* (%)					
	Yes	45 (12.4)	9 (9.8)	36 (13.3)	0.38
Median (IQR) CD4 count (cells/µL), *N* = 357		278 (111, 512)	298 (150, 459)	271(104, 549)	0.93
Median (IQR) viral load (log _10_ RNA copies/mL), *N* = 345		4.83 (3.71, 5.42)	4.78 (3.68, 5.36)	4.87 (3.71, 5.46)	0.44
Median (IQR) BMI (kg/m^2^), *N* = 361		22.3 (19.4, 26.0)	23.4 (19.6, 28.0)	21.9 (19.3, 25.1)	**0.006**
Median (IQR) eGFR (mL/min/1.73m^2^), *N* = 345		111.2 (90.2, 134.3)	101.2 (79.4, 126.9)	113.6 (92.7, 138.8)	**0.003**
^b^CKD staging, *N* = 345					
	CKD stage 1	262 (75.9)	54 (62.8)	208 (80.3)	**0.003**
	CKD stage 2	65 (18.8)	27 (31.4)	38 (14.7)	
	CKD stage 3	18 (5.2)	5 (5.8)	13 (5.0)	
Median (IQR) sCD14 (pg/nL)		5.53 (3.90, 8.57)	5.45 (3.82, 8.01)	5.64 (4.01, 8.69)	0.58
Median (IQR) sP-selectin (pg/nL)		0.12 (0.06, 0.19)	0.13 (0.07, 0.19)	0.11 (0.06, 0.20)	0.51
Median (IQR) CD4 + CD38 + HLA-DR+ (%)		1.05 (0.50, 2.21)	1.71 (0.74, 2.80)	1.48 (0.63, 3.26)	0.69
Median (IQR) CD8 + CD38 + HLA-DR+ (%)		1.88 (1.17, 3.27)	2.40 (1.20, 3.98)	2.28 (1.30, 4.00)	0.76
Median (IQR) CD4 + PD-1+ (%)		30.20 (21.30, 40.35)	31.25 (21.60, 41.45)	30.50 (21.40, 41.43)	0.94
Median (IQR) CD8 + PD-1+ (%)		33.90 (25.28, 47.33)	34.75 (26.88, 45.30)	34.10 (24.05, 45.83)	0.68
Median (IQR) monocyte count (X 10^3^ cell/µL), *N* = 354		0.47 (0.36, 0.61)	0.47 (0.35, 0.59)	0.47 (0.36, 0.62)	0.70
Median (IQR) lymphocyte count (X 10^3^ cell/µL), *N* = 354		1.50 (1.08, 2.01)	1.49 (1.21, 1.93)	1.49 (1.01, 2.04)	0.69
Median (IQR) platelet count (X 10^3^ cell/µL), *N* = 355		227.0 (175.3, 287.0)	220.0 (173.0, 281.0)	230.0 (174.3, 294.8)	0.63

Abbreviations: IQR = interquartile range; CVDs = cardiovascular diseases; BMI = body mass index; eGFR = estimated glomerular filtration rate; CKD = chronic kidney disease

Notes: *number does not add to total because of missing BP readings in two participants, ^a^ (male ≥ 45 years, female ≥ 55 years), ^b^ (CKD stage 1 = eGFR ≥ 90 mL/min/1.73m^2^, CKD stage 2 = eGFR (60–89) mL/min/1.73m^2^, CKD stage 3 = eGFR (30–59) mL/min/1.73m^2^

### Prevalence and predictors of hypertension

The prevalence of hypertension was 92/363 (25.3%, 95% CI 20.9–29.8%). Among all participants, the mean Systolic BP ± SD was 123.3 ± 17.3 mmHg and the median Diastolic BP (IQR) was 74.0 (70.0, 81.5) mmHg.

In univariable and multivariable analyses, none of the immune and platelet activation markers predicted the occurrence of HTN. Regarding traditional risk factors for CVDs, those participants who were overweight or obese had 69% more occurrence of HTN (aPR 1.69 (95% CI 1.17–2.44) compared to those participants who had underweight or normal weight. Additionally, the participants in CKD stage 2 had 97% more occurrence of HTN (aPR 1.97 (95% CI 1.34–2.88) compared to participants in CKD stage 1 (Table 2).

**Table 2:** Predictors of hypertension among HIV-infected treatment naïve individuals initiating ART

Predictor		number (%)	Univariable analysis			Multivariable analysis		
			cPR	95% CI	*p*- value	aPR	95% CI	*p*- value
CD4 + CD38 + HLA-DR+								
	3rd tertile	25 (21.7)	0.99	0.61–1.64	0.99	1.03	0.58–1.82	0.92
	2nd tertile	37 (31.1)	1.43	0.92–2.22	**0.12**	1.38	0.84–2.26	0.20
	1st tertile	24 (21.8)	1			1		
CD8 + CD38 + HLA-DR+								
	3rd tertile	29 (24.8)	0.94	0.61–1.46	0.79	-	-	-
	2nd tertile	30 (25.9)	0.98	0.64–1.52	0.94	-	-	-
	1st tertile	31 (26.3)	1			-		
CD4 + PD-1+								
	3rd tertile	29 (24.6)	0.96	0.62–1.49	0.85	-	-	-
	2nd tertile	31 (26.5)	1.03	0.64–1.59	0.88	-	-	-
	1st tertile	30 (25.6)	1			-		
CD8 + PD-1+								
	3rd tertile	30 (25.6)	1.24	0.77–1.99	0.37	1.45	0.82–2.56	0.20
	2nd tertile	36 (30.3)	1.46	0.93–2.29	**0.10**	1.53	0.94–2.51	0.09
	1st tertile	24 (20.7))	1			1		
sCD14								
	3rd tertile	27 (22.5)	0.81	0.52–1.26	0.35	-	-	-
	2nd tertile	29 (24.4)	0.88	0.57–1.35	0.56	-	-	-
	1st tertile	33 (27.7)	1			-		
sP-selectin								
	3rd tertile	31 (26.3)	1.26	0.80- 2.00	0.33	-	-	-
	2nd tertile	33 (27.5)	1.32	0.84–2.08	0.23	-	-	-
	1st tertile	25 (20.8)	1			-		
Viral load (RNA copies/mL)								
	≥ 1000	72 (24.9)	1.05	0.59–1.86	0.88	-	-	-
	50–999	4 (28.6)	1.20	0.45–3.23	0.72	-	-	-
	< 50	10 (23.8)	1			-		
CD4 count (cells/µL)								
	< 200	33 (23.1)	0.94	0.62–1.43	0.78	-	-	-
	200–350	22 (31.0)	1.27	0.81–1.99	0.31	-	-	-
	> 350	35 (24.5)	1			-		
CKD staging								
	CKD stage 3	5 (27.8)	1.35	0.62–2.95	0.45	1.49	0.67–3.29	0.33
	CKD stage 2	27 (41.5)	2.02	1.39–2.93	**< 0.001**	1.97	1.34–2.88	**0.001**
	CKD stage 1	54 (20.6)	1			1		
^a^Risky age for CVDs								
	Yes	19 (32.2)	1.34	0.88–2.04	**0.17**	1.30	0.83–2.06	0.26
	No	73 (24.0)	1			1		
Sex								
	Male	39 (30.2)	1.34	0.94–1.90	**0.11**	1.33	0.89–1.97	0.16
	Female	53 (22.6)	1			1		
BMI								
	^d^Overweight/^e^Obesity	39 (35.5)	1.68	1.19–2.38	**0.003**	1.69	1.17–2.44	**0.01**
	^b^Under/^c^Normal weight	53 (21.1)	1			1		
Cigarette smoking								
	Ever smoked	11 (19.3)	0.73	0.42–1.28	0.27	-	-	-
	Never smoked	81 (26.5)	1			-		
Alcohol consumption								
	Ever consumed	42 (24.3)	0.92	0.65–1.32	0.66	-	-	-
	Never consumed	50 (26.3)	1			-		
Family history of CVDs								
	Yes	13 (26.5)	1.06	0.64–1.75	0.64	-	-	-
	No	79 (25.2)	1			-		
Diabetes mellitus								
	Yes	2 (66.7)	2.67	1.78–6.05	**0.02**	2.34	0.86–6.36	0.09
	No	90 (25.0)	1			1		
Dyslipidaemia								
	Yes	47 (32.6)	1.24	0.56–2.73	0.59	-	-	-
	No	5 (26.3)	1			-		

Abbreviations: cPR = crude prevalence ratio; aPR = adjusted prevalence ratio; CI = confidence interval; sCD14 = soluble CD14; sP-selectin = soluble *P* selectin; CKD = Chronic Kidney Disease; CVDs = cardiovascular diseases; BMI = body mass index;

Notes: ^a^ (male ≥ 45 years, female ≥ 55 years); ^b^ BMI (< 18.5) kg/m^2^; ^c^ BMI = (18.5 to 24.9) kg/m^2^; ^d^ BMI = (25.0 to 29.9) kg/m^2^; ^e^ (BMI ≥ 30.0) kg/m^2^

## Discussion

The current study examined the relationship between HTN and both soluble and cellular markers of IA (sCD14, HLA-DR^+^CD38^+^ on CD4^+^ and CD8^+^ T cells) and exhaustion (PD-1^+^ on CD4^+^ and CD8^+^ T cells) and marker of platelet activation (sP-selectin) in ART naïve PLHIV who were starting ART. HTN is a problem among PLHIV with a reported prevalence higher than HIV-uninfected population [9, 10]. Understanding its pathogenesis is essential for potential preventive and therapeutic interventions. The established traditional risk factors for HTN cannot solely explain the increased risk of HTN in PLHIV. There is evidence to suggest that HIV-related factors, ART, and chronic IA may play a role [9, 19].

Our study involved a cross-sectional analysis of the baseline data of 365 participants of a clinical trial to determine the effect of low-dose ASA on HIV disease progression among HIV-infected individuals initiating ART. A quarter of the participants were hypertensive. While the median values of IA (excluding monocyte activation which was lower) and exhaustion markers and platelet activation marker were higher in hypertensive participants, none of these markers was found to significantly predict HTN.

In this study, there was no statistically significant difference in the median value of the marker of monocyte activation, sCD14 between participants with HTN and those without HTN. In fact, a lower median value of sCD14 was observed in the hypertensive participants. Our findings are in keeping with those of a study also conducted in East Africa - Uganda whereby sCD14 was not associated with incident HTN and the relationship between sCD14 and incident HTN was inverse [33]. Despite the similarity in findings, the Ugandan study was among ART-exposed PLHIV on six months of therapy [33]. This is not surprising, as previous studies have shown that even with successful treatment with ART the higher-than-normal levels of sCD14 in PLHIV persist or decrease but do not normalise [21, 24, 34].

Although the expression of sCD14 has a genetic basis [35], other studies conducted in Europe, Australia and the US also, among ART-exposed PLHIV, reported no significant association between sCD14 and HTN and/or BP parameters [34, 36–38]. On the contrary, a Norwegian study found higher levels of sCD14 among hypertensive PLHIV compared to non-hypertensive PLHIV. Furthermore, the study found sCD14 to be an independent predictor of only DBP but not SBP [39]. These reports altogether indicate that data on the association between sCD14 and HIV-associated HTN is still conflicting regardless of ART status and/or ethnicity. Further studies are required to establish convincingly the actual relationship between sCD14 and HIV-associated HTN.

The role of activated and/ or exhausted T lymphocytes in the pathogenesis of non-AIDS complications such as HTN among PLHIV has not been extensively studied. Contradictory to our hypothesis, we found that T lymphocyte activation and exhaustion did not predict HTN among our study participants. Similarly, T cell activation was unpredictive of HTN in a US-based study among African Americans and Hispanics untreated and treated HIV-infected women [40]. Two additional studies were also conducted in the US: one exploring the relationship between T cell activation and exhaustion and non-AIDS defining events generally; while the other looked at the relationship specifically with HTN [38, 41]. The former found no association between T cell activation markers and non-AIDS defining events including stroke. However, in this study marker of CD4^+^ T cell exhaustion had an association with the non-AIDS defining events that became insignificant after adjusting for CD4^+^ T cell count [41]. The latter study conducted among ART-exposed virologically suppressed PLHIV reported no association between dual expression of CD38 and HLA-DR antigens or expression of PD-1 on CD4 ^+^ and CD8 ^+^ T cells and HTN [38]. However, in this study, HTN was associated with lower CD4 ^+^ but not CD8 ^+^ T cells expressing CD38 singly. Drawing from our findings and these previous reports, T cell activation and exhaustion may not have a role in the pathophysiology of HTN in PLHIV of different races, gender, HIV viraemia and/or ART status. However, more evidence needs to be gathered from larger multi-centre studies to come to this conclusion as available reports are scant.

Our study was not primarily designed to study the relationship between HTN and IA and exhaustion among PLHIV. We conducted a cross-sectional analysis of available baseline data of clinical trial participants aiming at determining the effect of low-dose ASA among treatment naïve PLHIV initiating ART. The data, although large, may be not sufficient to establish the relationship between HTN and the studied markers of IA and exhaustion. Additionally, our study explored a narrow range of both soluble and cellular markers of IA and exhaustion. However, these markers were selected based on their reported roles in the pathogenesis of CVDs in the general population and/or experimental animals.

## Conclusion and recommendations

Although markers of IA and exhaustion were higher in hypertensive PLHIV among our study participants, they did not significantly predict HTN. In this study, only traditional risk factors for CVDs specifically CKD staging, and BMI significantly predicted the occurrence of HTN. Further larger multi-centric studies with a wider range of IA markers are needed to establish the relationship between HTN and immune activation and exhaustion among PLHIV.

### Electronic supplementary material

Below is the link to the electronic supplementary material.

Supplementary Material 1

Supplementary Material 2

## Data Availability

All data generated or analysed during this study are included in this published article [and its supplementary files].

## Abbreviations

AMI: Acute Myocardial Infarction
ART: Antiretroviral Therapy
ASA: Aspirin
BMI: Body Mass Index
BP: Blood Pressure
CKD: Chronic Kidney Disease
CRFs: Case Report Forms
CTCs: Care and Treatment Centers
CV: Cardiovascular
CVDs: Cardiovascular Diseases
DBP: Diastolic Blood Pressure
DM: Diabetes Mellitus
DTG: Dolutegravir
eGFR: Estimated Glomerular Filtration Rate
FBP: Full Blood Picture
HDL: C-high-density lipoprotein cholesterol
HTN: Hypertension
IA: Immune Activation
IL: Immunology Laboratory
IQR: Interquartile Range
LDL: C-low-density lipoprotein Cholesterol
MCRL: MUHAS Clinical Research Laboratory
IDC: Infectious Disease Centre Laboratory
MRRH: Mwananyamala Regional Referral Hospital
MRTH: Mbagala Rangi Tatu Hospital
MUHAS: Muhimbili University of Health and Allied Sciences
NIMR: National Institute for Medical Research
PBMCs: Peripheral Blood Mononuclear Cells
PLHIV: People Living with HIV and/or AIDS
SBP: Systolic Blood Pressure
sCD14: Soluble CD14
SD: Standard Deviation
sP: Selectin-soluble P-selectin
SPSS: Statistical Software for Social Sciences
SSA: Sub-Saharan Africa
TC: Total Cholesterol
TG: Triglycerides
TRRH: Temeke Regional Referral Hospital
