# Supplementary material for: Hypertension and immune activation in antiretroviral therapy naïve people living with human immunodeficiency virus
Source: BMC Infect Dis. 2024 Jun 24;24:630. doi: 10.1186/s12879-024-09548-x (PMC11197211; doi:10.1186/s12879-024-09548-x)
Supplement: Supplementary file 2 — Supplementary Material 2 [file 12879_2024_9548_MOESM2_ESM.docx]

Supplementary file 2- Socio-demographic and clinical characteristics of HIV-infected treatment naïve individuals initiating ART overall (*N=363) and by hypertension status.

| **Variable** | | **Total participants number (%)** | **Hypertensive**  **number (%)** | **Normotensive**  **number (%)** | **p-value** |
| --- | --- | --- | --- | --- | --- |
| Duration of HIV diagnosis, N=362 | |  |  |  |  |
|  | Less or equal to two weeks | 345 (95.3) | 90 (97.8) | 255 (94.4) | 0.26 |
|  | More than two weeks | 17 (4.7) | 2 (2.2) | 15 (5.6) |  |
| Site of recruitment, n (%) | |  |  |  |  |
|  | TRRH | 28 (7.7) | 4 (4.3) | 24 (8.9) | 0.22 |
|  | MRTH | 233 (64.2) | 65 (70.7.) | 168 (62.0) |  |
|  | MRRH | 102 (28.1) | 23 (25.0) | 79 (29.2) |  |
| Level of education, n (%) | |  |  |  |  |
|  | Informal | 26 (7.2) | 8 (8.7) | 18 (6.6) | 0.74 |
|  | Primary level | 215 (59.2) | 52 (56.5) | 163 (60.1) |  |
|  | Secondary level | 97 (26.7) | 27 (29.3) | 70 (25.8) |  |
|  | University or college | 25 (6.9) | 5 (5.4) | 20 (7.4) |  |
| CD4 count (cells/µL), N=357 | |  |  |  |  |
|  | < 200 | 143 (40.1) | 33 (36.7) | 110 (41.2) | 0.44 |
|  | 200-350 | 71 (19.9) | 22 (24.4) | 49 (18.4) |  |
|  | > 350 | 143 (40.1) | 35 (38.9) | 108 (40.4) |  |
| Viral load (RNA copies/mL), N=345 | |  |  |  |  |
|  | < 50 | 42 (12.2) | 10 (11.6) | 32 (12.4) | 0.93 |
|  | 50-999 | 14 (4.1) | 4 (4.7) | 10 (3.9) |  |
|  | ≥ 1000 | 289 (83.8) | 72 (83.7) | 217 (83.8) |  |
| ^a^Risky Age for CVDs | |  |  |  |  |
|  | Yes | 59 (16.3) | 19 (20.7) | 40 (14.8) | 0.19 |
|  | No | 304 (83.7) | 73 (79.3) | 231(85.2) |  |
| BMI (kg/m^2^), N=361 | |  |  |  |  |
|  | ^b^Underweight /^c^Normal weight | 251 (69.5) | 53 (57.6) | 198 (73.6) | **0.004** |
|  | ^d^Overweight /^e^Obesity | 110 (30.5) | 39 (42.4) | 71 (26.4) |  |
| Cigarette smoking | |  |  |  |  |
|  | Ever smoked | 57 (15.7) | 11 (12.0) | 46 (17.0) | 0.25 |
|  | Never smoked | 306 (84.3) | 81 (88.0) | 225 (83.0) |  |
| Alcohol consumption | |  |  |  |  |
|  | Ever consumed | 173 (47.7) | 42 (45.7) | 131 (48.3) | 0.66 |
|  | Never consumed | 190 (52.3) | 50 (54.3) | 140 (51.7) |  |
| Diabetes Mellitus | |  |  |  |  |
|  | Yes | 3 (0.8) | 2 (2.2) | 1 (0.4) | 0.16 |
|  | No | 360 (99.2) | 90 (97.8) | 270 (99.6) |  |
| Family history of CVDs | |  |  |  |  |
|  | Yes | 49 (13.5) | 13 (14.1) | 36 (13.3) | 0.84 |
|  | No | 314 (86.5) | 79 (85.9) | 235 (86.7) |  |
| Dyslipidaemia, N= 163 | |  |  |  |  |
|  | Yes | 144 (88.3) | 47 (90.4) | 97 (87.4) | 0.58 |
|  | No | 19 (11.7) | 5 (9.6) | 14 (12.6) |  |
| ^f^CKD staging, N=345 | |  |  |  |  |
|  | CKD stage 1 | 262 (75.9) | 54 (62.8) | 208 (80.3) | **0.003** |
|  | CKD stage 2 | 65 (18.8) | 27 (31.4) | 38 (14.7) |  |
|  | CKD stage 3 | 18 (5.2) | 5 (5.8) | 13 (5.0) |  |
| History of bacterial infection at enrolment | |  |  |  |  |
|  | Yes | 69 (19.0) | 9 (9.8) | 60 (22.1) | **0.009** |
|  | No | 294 (81.0) | 83 (90.2) | 211 (77.9) |  |
| History of use of anti-inflammatory drug within past month | |  |  |  |  |
|  | Yes | 45 (12.4) | 9 (9.8) | 36 (13.3) | 0.38 |
|  | No | 318 (87.6) | 83 (90.2) | 235 (86.7) |  |
| Monocyte status, N=354 | |  |  |  |  |
|  | Monocytosis (Monocyte count > 0.70x10^3^ cell/µL) | 54 (15.3) | 9 (10.3) | 45 (16.9) | 0.14 |
|  | Normal monocyte count (Monocyte count $\leq$ 0.70x10^3^ cell/µL) | 300 (84.7) | 78 (89.7) | 222 (83.1) |  |
| Lymphocyte status, N=354 | |  |  |  |  |
|  | Lymphocytosis (Lymphocyte count > 3.70x10^3^ cell/µL) | 8 (2.3) | 1 (1.1) | 7 (2.6) | 0.06 |
|  | Normal lymphocyte count (Lymphocyte count (1.00- 3.70) x 10^3^ cell/µL) | 277 (78.2) | 76 (87.4) | 201 (75.3) |  |
|  | Lymphopenia (Lymphocyte count < 1.00x10^3^ cell/µL) | 69 (19.5) | 10 (11.5) | 59 (22.1) |  |
| Platelet status, N=355 | |  |  |  |  |
|  | Thrombocytopenia (Platelet count < 150x10^3^ cell/µL) | 66 (18.6) | 15 (17.2) | 51 (19.0) | 0.71 |
|  | Normal platelet count (Platelet count $\geq$  150x10^3^ cell/µL) | 289 (81.4) | 72 (82.8) | 217 (81.0) |  |

Abbreviations: IQR= interquartile range; CVDs= cardiovascular diseases; BMI= body mass index; CKD= chronic kidney disease

Notes: *number does not add to total because of missing BP readings in two participants, ^a^ (male ≥ 45 years, female ≥ 55 years); ^b^ BMI (< 18.5) kg/m^2^; ^c^ BMI = (18.5 to 24.9) kg/m^2^; ^d^ BMI = (25.0 to 29.9) kg/m^2^; ^e^ (BMI ≥ 30.0); ^f^ (CKD stage 1 = eGFR ≥ 90 mL/min/1.73m^2^, CKD stage 2 = eGFR (60- 89) mL/min/1.73m^2^, CKD stage 3= eGFR (30- 59) mL/min/1.73m^2^.
